# Phase II study of capecitabine and mitomycin C as first-line treatment in patients with advanced colorectal cancer

**DOI:** 10.1038/sj.bjc.6602039

**Published:** 2004-07-20

**Authors:** S Rao, D Cunningham, T Price, M E Hill, P J Ross, N Tebbutt, A R Norman, J Oates, P Shellito

**Affiliations:** 1 Department of Medicine, Royal Marsden Hospital, Downs Road, Sutton, Surrey SM2 5PT, UK; 2 Queen Elizabeth Hospital, Adelaide, South Australia

**Keywords:** colorectal cancer, advanced, chemotherapy, capecitabine, mitomycin C

## Abstract

This study was designed to assess the safety and efficacy of capecitabine and mitomycin C (MMC) in previously untreated patients with advanced colorectal cancer (CRC). Patients received capecitabine 2500 mg m^2^ day 1, orally divided in two doses of 1250 mg m^−2^ in the morning and evening for 14 days every 21 days and MMC 7 mg m^−2^ (maximum total dose 14 mg) as an intravenous bolus every 6 weeks for a total of four courses. The median age was 70 years (range 24–85) and the majority of patients (86.9%) were of performance status 1/2. The most common metastatic site was liver. In all, 84 patients were assessable for response. The overall response rate was 38% (95% CI: 27.7–49.3) and a further 33.3% of patients achieved stable disease over 12 weeks. There was good symptom resolution ranging from 64 to 86%. Grade 3/4 toxicity was as follows: hand–foot syndrome 19.7%; diarrhoea 10%; neutropenia 2.4%; infection 2.3%. Capecitabine and MMC have shown encouraging activity with a favourable toxicity profile, a convenient administration schedule, and could be considered for patients deemed unsuitable for oxaliplatin and irinotecan combinations.

Colorectal cancer (CRC) is the second most common cancer in Europe and 500 000 patients die from the disease annually worldwide (Parkin *et al*, 2001a, 2001b). Although 70–80% of new cases of colon cancer undergo potentially curative surgery, 40% develop recurrent or metastatic disease. For many years, fluorouracil (5FU) has been the backbone of treatment for advanced CRC.

Intravenous bolus injections of 5FU yielded overall response rates of 10% and a median overall survival (OS) of 11 months. These results were improved by prolonged infusion of 5FU, which led to higher response rates, a different toxicity profile and a small but significant increase in OS to 12 months (Meta-analysis Group In Cancer, 1998). A variety of modulating agents have been investigated in combination with 5FU. Leucovorin is the most widely used agent and has demonstrated an improved 1-year survival when compared to 5FU alone (48% compared to 43%, respectively, HR 0.88; 95% CI: 0.81–0.96; *P*=0.003) (Piedbois and Michiels, 2003).

Oxaliplatin and irinotecan are agents that have improved the outcome in advanced CRC in the last decade. They have been combined with infused and bolus schedules of 5FU. In two large randomised studies, irinotecan paired with either bolus 5FU/LV (IFL) or infused 5FU/LV demonstrated an improved OS compared to 5FU/LV alone (Douillard *et al*, 2000; Saltz *et al*, 2000). A randomised study of oxaliplatin combined with infused 5FU/LV *vs* 5FU/LV alone produced superior response rates and progression-free survival (PFS) for the combination arm. However, the improvement in OS was not statistically significant (de Gramont *et al*, 2000). Recently, a US Intergroup study N9741 demonstrated an OS benefit with infused 5FU/LV/oxaliplatin (FOLFOX4) compared to IFL and irinotecan/oxaliplatin (Goldberg *et al*, 2004). However, 60% of patients receiving FOLFOX 4 crossed over to irinotecan on disease progression, whereas only 24% in the IFL arm received second-line oxaliplatin.

Furthermore, there have been concerns over the safety of these combination regimens, in particular the IFL regimen. Higher than expected 60-day mortality rates were reported for this regimen in the above Intergroup N9741 study and the CALGB C89803 adjuvant study due to a gastrointestinal syndrome (diarrhoea, vomiting, dehydration and neutropenia) and vascular syndrome (acute fatal myocardial infarction, cerebrovascular accident and pulmonary embolism) (Rothenberg *et al*, 2001).

The oral fluoropyrimidines are another class of drug to have been implemented in the last decade. Capecitabine is an oral tumour-selective fluoropyrimidine and thus generates 5FU preferentially at the tumour site. The final conversion step to 5FU is dependent on thymidine phosphorylase, which is significantly more active in tumour than normal tissue, hence the specific targeting of 5FU (Miwa *et al*, 1998; Schuller *et al*, 2000). Capecitabine has been evaluated in two phase III studies in this setting, employing bolus 5FU/LV as the control arm. Equivalent times for disease progression and OS were observed in both arms, and the toxicity profile for capecitabine was notably different with significantly less diarrhoea, nausea, stomatitis and alopecia than bolus 5FU/LV (*P*<0.001) (Twelves, 2002).

In the United Kingdom, the National Institute for Clinical Excellence (NICE) has issued guidance on the treatment of advanced CRC based on the currently available data. It has recommended 5FU/LV in combination with oxaliplatin as first-line treatment for patients with potentially resectable metastatic disease confined to the liver. For all other patients, first-line chemotherapy should be instituted with capecitabine monotherapy or 5FU/LV. Furthermore, a recent physician-based survey of first-line treatment for advanced CRC revealed that 30% of patients worldwide still receive fluoropyrimidine monotherapy (Capecitabine Usage Tracking Study. The Research Partnership, 2004).

Mitomycin C (MMC) has been used in the treatment of CRC for many years. A randomised study of protracted venous infusion 5FU with or without MMC conducted in this setting resulted in an improved response rate, failure-free survival (FFS) and better quality of life, but no OS benefit for the combination arm (Ross *et al*, 1997). MMC has also been shown to upregulate intratumoural thymidine phosphorylase activity, which may increase synergism with capecitabine (Sawada *et al*, 1998).

The widespread use of fluoropyrimidine monotherapy in the UK and the potentially increased synergism of capecitabine with MMC led to this study design to provide a more efficacious treatment without compromising tolerability. The aim of the study was to assess the safety and efficacy of capecitabine in combination with MMC in previously untreated patients with advanced CRC.

## MATERIALS AND METHODS

### Eligibility

This open-label nonrandomised phase II study was conducted in two centres in the United Kingdom and Australia. Written informed consent was obtained from all patients. The local research and ethics committee approved the study.

The eligibility criteria were histologically confirmed advanced colorectal adenocarcinoma, no prior chemotherapy except adjuvant treatment at least 6 months previously, adequate bone marrow (platelets>100 × 10^9^ l^−1^, white count >3 × 10^9^ l^−1^), renal (creatinine clearance >30 ml min^−1^) and hepatic (<1.5 × the upper limit of normal range) function, WHO performance status 0–2, life expectancy of at least 3 months and no concurrent uncontrolled medical illness. Patients were excluded if there were medical or psychiatric conditions precluding informed consent, renal impairment, known malabsorption syndrome or significant cardiac disease, arrythmias or angina pectoris.

### Therapy

Capecitabine, 2500 mg m^2^ day 1, was given orally divided in two doses of 1250 mg m^−2^ in the morning and evening for 14 days, followed by a 7-day treatment-free interval with each cycle of treatment being repeated every 21 days. MMC, 7 mg m^−2^ (maximum total dose 14 mg), was given as an intravenous bolus every 6 weeks for a total of four courses. Patients continued on treatment for 12 weeks and were then re-assessed. If there was no disease progression, treatment continued for a further 12 weeks.

### Toxicity evaluation and dose modification

Toxicity was evaluated and graded according to the National Cancer Institute common toxicity criteria (version 2.0) (Trotti *et al*, 2000). For grade 3 nonhaematological toxicity, capecitabine treatment was suspended until resolution and re-initiated with 25% dose reduction for the first appearance and 50% for the second. For grade 4 nonhaematological toxicity, capecitabine therapy was either terminated or suspended until resolution, with a 50% dose reduction on re-initiation at the treating physician's discretion. For haematological toxicity, if the absolute neutrophil count <1.0 × 10^9^ l^−1^ or the platelet count <100 × 10^9^ l^−1^, capecitabine and MMC were delayed until resolution and re-initiated at full dose for 1 week delay and with a subsequent 25% dose reduction for 2 week delay.

### Safety evaluation

Patients were assessed at baseline with a full medical history and physical examination including PS, full blood count, serum biochemistry including electrolytes, hepatic and renal function tests and serum carcinoembryonic antigen (CEA). During the study, full blood count, urea and electrolytes, liver function tests and CEA were performed at weeks 3 and 6 initially, then 6 weekly thereafter.

### Efficacy evaluation

Tumour response by CT assessment was performed according to RECIST criteria at 12 and 24 weeks (Therasse *et al*, 2000). FFS and OS were calculated for all patients from the date of treatment initiation to the date of disease progression or death, respectively (intent-to-treat analysis), using the Kaplan–Meier method. Patients still alive were censored at the date of last contact.

### Statistical methods

This phase II study was conducted according to the Simon optimal two-stage design. The sample size was calculated with 90% power to detect an objective response rate of 50% and rule out a lower limit of 25% of this estimate using a one-sided alpha of 0.05. In all, 17 patients were enrolled in the first stage; following this, an interim analysis was performed. As there were more than five responding patients, the study proceeded to the second stage and an additional 20 patients were accrued. After a total of 37 patients, there were more than 13 responding patients; thus, ethics approval was sought for a total cohort of 100 patients in order to obtain a tighter estimate of the response rate (95% CI±10%).

## RESULTS

A total of 92 patients were recruited between September 2001 and January 2004. Patient demographics are shown in Table 1

**Table 1:** Patient demographics

**Patient characteristics**	**Number**
Total number entered	92
	
Age median (range)	70 (24–85)
Male/female	55/37
	
*ECOG performance status*	
0	30 (32.6%)
1	50 (54.3 %)
2	10 (10.9%)
Unknown	2 (2.1%)
	
*Primary site*	
Rectum	29 (31.5%)
Colon	63 (68.5%)
	
*Original Dukes stage*	
A	1
B	12
C	27
D	29
Unknown	23
	
*Metastatic sites*	
Liver	62
Lung	34
Nodal	21
Peritoneum	8
Locoregional	11
	
*Histological differentiation*	
Well	2
Moderate	68
Moderate–poorly	2
Poorly	12
Unknown	8

. The median age was 70 (range 24–85) years, the majority of patients were performance status (PS) 1 and the most common metastatic disease site was liver.

### Chemotherapy delivery

All eligible patients commenced treatment at full dosage for a planned 24-week course. The median treatment duration was 16 weeks (range 1–24). Dose intensities of the intended starting dose of capecitabine and MMC were 82 and 78%, respectively. Treatment delays occurred in 38 (43.7%) patients.

### Tumour response and symptomatic response

In all, 84 patients were evaluable for response at the time of analysis. Eight patients were not evaluable for response: four died prior to response assessment, one had nonmeasurable disease, one patient underwent abdominal surgery after one cycle and two patients have not yet had response assessment at 12 weeks. Response data are shown in Table 2

**Table 2:** Response to capecitabine/MMC

**Best response**	**No of patients (*N*=84)**	**% of assessable patients**
CR	5	5.95
PR	27	32.1
SD	28	33.33
PD	24	28.57
NE^*^	8	

NE=nonevaluable; CR=complete response; PR=partial response; SD=stable disease; PD=progressive disease.

. There were five complete responses (CRs) and 27 partial responses (PRs) (overall response rate 38; 95% CI 27.7–49.3); this represents the best-achieved response rate.

In total, 28 patients (33.3%) achieved stable disease for a minimum of 12 weeks and 24 (28.5%) developed progressive disease. Symptomatic improvement was observed in a large proportion of patients as shown in Table 3

**Table 3:** Symptomatic response

**Symptom**	**Number**	**%**
Pain	18/31	58.1
Anorexia	12/15	80.0
Weight loss	13/15	86.7
Bowel	20/25	80.0

. The bowel symptoms were in patients with a primary cancer *in situ* and synchronous metastases.

### Toxicity

Overall, the treatment was well tolerated and the most commonly reported events are demonstrated in Table 4

**Table 4:** Toxicity

**Toxicity**	**All grades (*N*=86) (%)**	**Grade 3/4 (*N*=86) (%)**
Diarrhoea	55.8	10.4
Stomatitis	26.7	0
Alopecia	3.7	0
Nausea	40.7	2.3
Infection	10.5	2.3
Hand–foot syndrome	54.7	19.7
Fever	5.8	1.2
		
	**(*N*=85) (%)**	**(*N*=85) (%)**
Anaemia	52.9	2.4
Neutropenia	39.3	2.4
Thrombocytopenia	22.4	4.7

. The most significant grade 3/4 toxicities were hand–foot syndrome (19.7%), diarrhoea (10%), thrombocytopenia (4.7%) and neutropenia (2.4%). There were no cases of grade 3/4 stomatitis and grade 2 alopecia did not occur with this regimen. There were no treatment-related deaths with this regimen.

Three (3.75%) patients developed red cell fragmentation and therefore discontinued MMC, but no cases of haemolytic uraemic syndrome were detected.

### Survival

**Figure 1:**
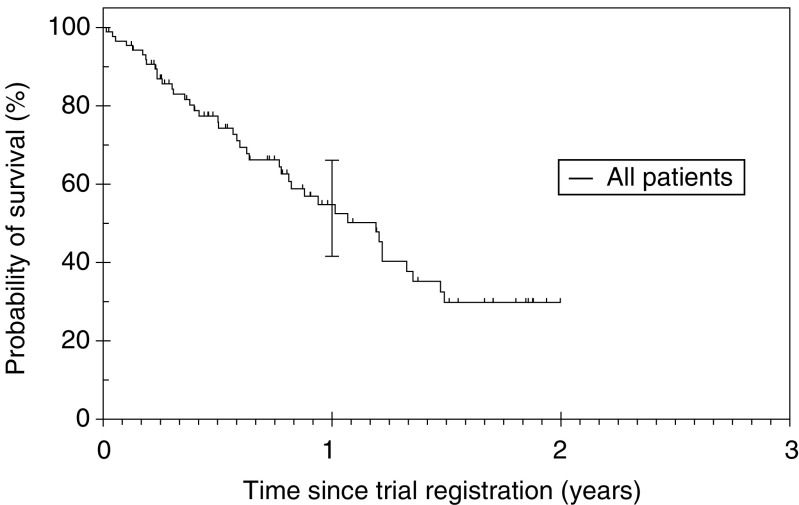
Overall survival (OS): Median OS was 14.3 months (95% CI 11.39–17.2).

**Figure 2:**
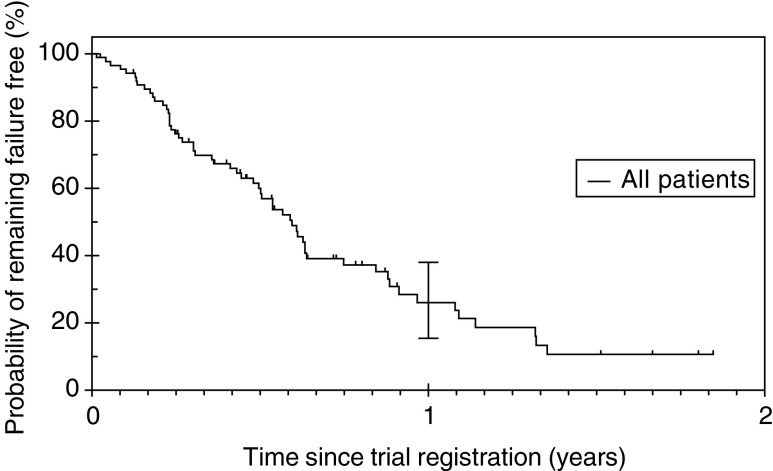
Failure-free survival (FFS): Median FFS was 7.11 months (95% CI 6.13–8.10).

With a median follow-up of 12.7 months, 55.4% of patients had died at the time of analysis. The median OS for this regimen was 14.3 months (95% CI 11.39–17.2) with 1 year OS of 54.8% (95% CI 41.6–66.1) (Figure 1

). The median FFS was 7.11 months (95% CI 6.13–8.10) with 1 year FFS of 26% (95% CI 15.4–38.0) (Figure 2

).

### Second-line treatment

In all, 29 patients (36.3%) received second-line chemotherapy on disease progression (10 irinotecan; two irinotecan/gefitinib; six capecitabine/oxaliplatin; one infused 5FU). Three patients were re-challenged with capecitabine/MMC and seven with capecitabine alone; these patients demonstrated a progression-free interval of ⩾6 months after an initial response to XMMC.

## DISCUSSION

This trial has demonstrated that capecitabine/MMC is an effective and well-tolerated regimen for patients with untreated advanced CRC. It produced an overall response rate of 38% (95% CI 27.7–49.3), median FFS of 7.11 months (95% CI 6.13–8.10) and a median OS of 14.3 months (95% CI 11.39–17.2). Furthermore, 33.3% of patients achieved stable disease and overall the regimen produced good symptom resolution. The median OS previously reported with capecitabine alone was 12.9 months (95% CI 12.0–14.0) and FFS 4.6 months (95% CI 4.3–5.3) (Twelves, 2002).

Grothey *et al* recently compared capecitabine with the addition of oxaliplatin or irinotecan in a randomised phase II study. The ORRs for capecitabine/irinotecan and capecitabine/oxaliplatin were 37 and 49.3%, and PFS 8.2 and 6.6 months, respectively (Grothey *et al*, 2003). A European phase II study of 43 patients evaluating capecitabine combined with oxaliplatin demonstrated an impressive ORR of 55% (95% CI 45–65) and median time to disease progression (PFS) of 7.4 months; however, there was one treatment-related death and 11% grade 3/4 sensory neuropathy (Tabernero *et al*, 2002). Furthermore, grade 3/4 diarrhoea and dehydration requiring hospitalisation in another phase II study evaluating capecitabine and oxaliplatin necessitated a dose reduction of capecitabine from 2000 to 1500 mg m^−2^ for 14 days every 21 days. At the lower dose of capecitabine (1500 mg m^−2^), the ORR was 37.1% (95% CI 21.5–55.1) and median PFS was 6.9 months with 20% grade 3/4 diarrhoea and one treatment-related death (Shields *et al*, 2004). A phase II study of 56 patients investigating capecitabine and irinotecan showed an ORR of 45% (95% CI 30–60), and although toxicity was manageable there were two toxic deaths (Munoz *et al*, 2003). Therefore, the addition of capecitabine to MMC may be less efficacious than these combinations, but is associated with a more acceptable toxicity profile.

However, the median OS of 14.3 months produced with this combination is somewhat disappointing, particularly compared to the 5FU/LV irinotecan and oxaliplatin combinations which have produced a median OS approaching 20 months (Goldberg *et al*, 2004). Nevertheless, this may be partly accounted for by a selection bias in this study – by comparison with other contemporaneous studies this was an older population (median age 70 years), who were considered unlikely to tolerate oxaliplatin or irinotecan combinations. This is also reflected in the use of second-line treatment which is relatively low – 36.3% compared to 71% in an audit conducted at our institute of patients who received initial fluoropyrimidine therapy as part of a tumour vaccine study (median age 63 years), and 67 and 75%, respectively, in the recent Intergroup N9471 study for the irinotecan and oxaliplatin arms.

There is relatively little information regarding chemotherapy in this setting for elderly patients. Our own data have demonstrated that they derive similar benefits to their younger counterparts from 5FU-based palliative chemotherapy (Popescu *et al*, 1999). Although it has been suggested that irinotecan and oxaliplatin regimens can be administered to the elderly, they can be associated with considerable toxicity. A recent retrospective study reported the tolerance and efficacy of irinotecan or oxaliplatin combination therapy in 66 elderly patients (age >74 years) with advanced CRC. In total, 44 and 22 patients received oxaliplatin or irinotecan, respectively, and the median age was 78 years. Overall, an ORR of 21.5%, median PFS 6.8 months and median OS 11.2 months were reported in the first-line setting. There was significant toxicity: 42% of patients experienced grade 3/4 toxicity; neutropenia 17%, diarrhoea 15%, neuropathy 11%, nausea 8% and thrombocytopenia 6% (Aparicio *et al*, 2003). In our study, 30 (32.6%) patients were ⩾74 years and there were relatively few grade 3/4 toxicities observed and minimal myelosuppression. Thus, it appears that capecitabine and MMC can be safely administered to elderly patients.

Despite the currently available data for chemotherapy in advanced CRC, there is still considerable variation in practice among the oncological community worldwide. Indeed, a recent physician-based survey conducted in several countries (including North America, Canada and parts of Europe) has demonstrated that a significant proportion of patients treated with first-line therapy still receive fluoropyrimidine monotherapy. In the UK, this figure is particularly high (59%), which may be explained by NICE guidance. However, the corresponding figures for North America, Canada and Germany are 30, 32 and 42%, respectively (Capecitabine Usage Tracking Study. The Research Partnership, 2004). This implies that there is a relatively large subgroup of patients with advanced CRC, who are deemed unsuitable for oxaliplatin or irinotecan combination therapy.

In summary, this trial has demonstrated that capecitabine and MMC is an active and safe combination in untreated advanced CRC. It provides ease of administration, while avoiding the potential indwelling venous catheter-related complications, and can be given in the outpatient setting. It may be a valuable therapeutic option for those patients considered unsuitable for irinotecan and oxaliplatin. Furthermore, it offers an alternative treatment for those patients receiving fluoropyrimidine monotherapy, in which context MMC improves efficacy without incurring additional toxicity.
